# Physiologic regulation of heart rate and blood pressure involves connexin 36–containing gap junctions

**DOI:** 10.1096/fj.201600919RR

**Published:** 2017-05-22

**Authors:** Varinder K. Lall, Gareth Bruce, Larysa Voytenko, Mark Drinkhill, Kerstin Wellershaus, Klaus Willecke, Jim Deuchars, Susan A. Deuchars

**Affiliations:** * Faculty of Biological Sciences, School of Biomedical Sciences, University of Leeds, Leeds, United Kingdom;; † Division of Cardiovascular and Diabetes Research, Institute of Cardiovascular and Metabolic Medicine, University of Leeds, Leeds, United Kingdom;; ‡ Life and Medical Sciences Institute (LIMES), University of Bonn, Bonn, Germany

**Keywords:** sympathetic, cardiovascular, spinal cord, knockout models

## Abstract

Chronically elevated sympathetic nervous activity underlies many cardiovascular diseases. Elucidating the mechanisms contributing to sympathetic nervous system output may reveal new avenues of treatment. The contribution of the gap junctional protein connexin 36 (Cx36) to the regulation of sympathetic activity and thus blood pressure and heart rate was determined using a mouse with specific genetic deletion of Cx36. Ablation of the Cx36 protein was confirmed in sympathetic preganglionic neurons of Cx36-knockout (KO) mice. Telemetric analysis from conscious Cx36 KO mice revealed higher variance in heart rate and blood pressure during rest and activity compared to wild-type (WT) mice, and smaller responses to chemoreceptor activation when anesthetized. In the working heart–brain stem preparation of the Cx36-KO mouse, respiratory-coupled sympathetic nerve discharge was attenuated and responses to chemoreceptor stimulation and noxious stimulation were blunted compared to WT mice. Using whole cell patch recordings, sympathetic preganglionic neurons in spinal cord slices of Cx36-KO mice displayed lower levels of spikelet activity compared to WT mice, indicating reduced gap junction coupling between neurons. Cx36 deletion therefore disrupts normal regulation of sympathetic outflow with effects on cardiovascular parameters.—Lall, V. K., Bruce, G., Voytenko, L., Drinkhill, M., Wellershaus, K., Willecke, K., Deuchars, J., Deuchars, S. A. Physiologic regulation of heart rate and blood pressure involves connexin 36–containing gap junctions.

Normal physiology requires precise regulation of the cardiovascular system, maintaining blood pressure and cardiac output within appropriate ranges in response to internal and external environmental changes. This maintenance is provided to a large extent by the autonomic nervous system (1). Dysfunction of the autonomic nervous system, and in particular increased activity of the sympathetic branch, is associated with development, maintenance, and progression of cardiovascular diseases such as hypertension (1) and heart failure (2). Recent interventions for cardiovascular diseases, which include renal nerve ablation, carotid sinus denervation, carotid body ablation, carotid sinus stimulation (3), and transcutaneous vagal nerve stimulation (4), all lead to reduced sympathetic outflow. An increased understanding of sympathetic nervous control can therefore offer new insights into the mechanisms underlying potential therapies and may reveal new targets for future therapies.

Recent advances using genetic modification for neuronal tracing and optogenetics have considerably advanced and consolidated knowledge of the central neuronal circuits underlying sympathetic control (5). Because sympathetic preganglionic neurons (SPNs) are the last point in the CNS where sympathetic outflow can be influenced, regulation of their activity therefore provides a means of controlling sympathetic activity.

The presence of gap junctions (GJs) between SPNs influences their activity because electronic coupling allows transfer of electrical signals rapidly and securely between coupled cells (5). Further, as 3 different gap junction blockers reduced patterned rhythmic network activity in the intermediolateral cell column (IML) of the spinal cord, where SPNs are located (6), electrical coupling through GJs may contribute to this activity. Because rhythmicity underlies sympathetic nerve activity (SNA) in normal cardiovascular control (7), elucidating the contribution of GJs in regulation of sympathetic activity and their influence on the control of the cardiovascular system would enhance our overall understanding of what affects sympathetic outflow.

GJs in SPNs include the connexin 36 (Cx36) GJ protein because Cx36 immunoreactivity has been detected between SPNs (8). Therefore, this study uses a multidisciplinary approach to investigate the roles of Cx36 in autonomic control. We show that deletion of Cx36 causes dysregulation of sympathetic outflow, cardiovascular variables, and their reflex control. This dysregulation is associated with disruption of normal GJ coupling in SPNs.

## MATERIALS AND METHODS

Experiments were performed under UK Home Office License and in accordance with the regulations of the UK Animals (Scientific Procedures) Act of 1986. Cx36flox cyan fluorescent protein (CFP) mice (MGI ID 2178050; MGI symbol B6;129P2-Gjd2^tm4Kwi^/Cnrm, EMMA ID EM:02510) were generated in the laboratory of K.W. (9) as a genetic mixture (50%) of the C57BL/6 and the 129 strain and then backcrossed to C57BL/6 so that they had 75% of C57BL/6 genetic background. For this study, Cx36flox(CFP) homozygotes were crossed with phosphoglycerate kinase I promoter (PGK)-Cre mice [MGI ID 2178050, MGI symbol: Tg(Pgk1-cre)1Ln], which resulted in excision of E1 and E2 in both alleles and knockout (KO) of Cx36 with expression of CFP ubiquitously. These mice are henceforth referred to as Cx36-KO and wild-type (WT) littermates from breeding with C57BL/6 were used as controls.

### PCR

Ear samples were digested and genomic DNA extracted by following the DNeasy Blood and Tissue kit (Qiagen, Germantown, MD, USA) or DirectPCR (Viagen Biotech, Los Angeles, CA, USA) protocols. These involved an overnight proteinase K digestion followed by heat inactivation of the enzyme and purification (DNeasy only). For DNA obtained by the DNeasy protocol, amplification and detection of the Cx36 gene was performed in a 25-µl reaction mixture containing 12.5 µl HotStarTaq Master Mix (Qiagen), 1.25 µl DMSO, 4.75 µl H_2_O, 5 µl DNA template, and 0.5 µl each of the primers Cx36USP-1 (5′-TAAGTGCAATAAAGGGGGAGGGCCTCG-3′), Cx36DSP-1 (5′-GAGACAGGAGAAGGTATTCCCAAGGGC-3′,) and DSP-CFP (5′-AAGAAGTCGTGCTGCTTCATGTGG-3′). PCR conditions were 95°C for 5 min, 41 cycles at 95°C for 45 s, 56°C for 45 s, 72°C for 1 min, finishing with 72°C for 10 min. This reaction mixture was modified by increasing the H_2_O to 8.75 µl to accommodate a lower volume (1 µl) of DNA template when using samples processed by the DirectPCR protocol. Amplicons obtained were a 311-bp product for the WT Cx36 allele and a 504-bp product for the Cx36-deleted allele. Heterozygotes were distinguishable by the presence of both bands.

### Immunohistochemistry

Because CFP levels in Cx36-KO reporter mice are insufficient to be visualized directly, immunohistochemical methods were used. Adult mice were deeply anesthetized with pentobarbital solution (60 mg/kg, i.p.) and transcardially perfused with 100 ml 0.1 M PBS (pH 7.4), followed by 200 ml 4% paraformaldehyde in 0.1 M phosphate buffer (pH 7). The spinal cord was removed from the animal and postfixed overnight. Coronal sections of spinal cord tissue were taken at 30 to 50 µm on a vibrating microtome (Leica, Milton Keynes, United Kingdom) and incubated with primary chicken anti–green fluorescent protein (GFP) (Abcam, Cambridge, MA, USA) or rabbit anti-GFP (Thermo Fisher Scientific, Waltham, MA, USA) and goat anti–choline acetyltransferase (ChAT) (1:500; EMD Millipore, Billerica, MA, USA). Alexa Fluor 555–conjugated donkey anti-chicken (1:1000; Thermo Fisher Scientific) or Alexa Fluor 555–conjugated donkey anti-rabbit (1:1000; Thermo Fisher Scientific) was used to localize and visualize the primary antibodies. Secondary antibodies were diluted in PBS and sections incubated for 3 h at room temperature. Sections were washed in PBS (3× for 10 min), mounted on glass slides in Vectashield (Vector Laboratories, Burlingame, CA, USA), and viewed under an Eclipse E600 epifluorescence microscope (Nikon, Tokyo, Japan). Images were obtained using an integrated CCD camera attached to an Acquis image-capture system (Synoptics, Cambridge, United Kingdom). CorelDRAW 17 (Corel, Ottawa, ON, Canada) was used to adjust the image brightness, contrast, and intensity, if required.

### Telemetry

#### Implantation of telemetric probes

Experiments were performed on female homozygous Cx36-KO mice and WT littermates (age 6–8 mo). Mice were anesthetized with isoflurane (Merial, Harlow, United Kingdom) (5% induction and 1.5–2% maintenance) and implanted with radiotelemetry probes (PA-C10; Data Sciences International, New Brighton, MN, USA), which allowed continual measurements of arterial blood pressure (ABP) (mmHg) and heart rate (beats per minute, bpm). Blood pressure and heart rate were measured from a catheter inserted into the carotid artery to the level of the aortic arch. The body of the probe was placed into a subcutaneous pocket, where it remained for the duration of the study. After closure of the wound, the mice were placed into a recovery chamber heated at 37°C until they recovered.

Mice were individually housed in their home cages in a temperature-controlled room set to a 12:12-h light–dark cycle with free access to food and water. After 7 d of recovery, the telemetry probes were switched on. Running wheels were introduced on d 3 after recovery.

#### Chemoreceptor stimulation

Mice were removed from cages, anesthetized with isoflurane (5% induction and 1.5–2% maintenance), and placed on a heated pad at 37°C. A catheter was inserted *via* a small incision into the jugular vein for intravenous drug administration. Peripheral chemoreceptors were activated by administration of NaCN (Thermofisher Acros Organics, Geel, Belgium) intravenously at 0.3% diluted in 0.9% saline solution, administered in an intravenous bolus of 10 µl. These recordings were conducted during daylight hours. Three trials were conducted per animal with responses averaged; at least 5 min of recovery was allowed between each trial.

#### Analysis

The average interbeat (R-R) interval (s), ABP (mmHg), and heart rate (bpm) in all mice were analyzed by using LabChart software (ADInstruments, Oxford, United Kingdom) during 24-h diurnal recordings or autonomic reflex stimulation. The average R-R interval (s), ABP (mmHg), and heart rate (bpm) were analyzed for 24 h with and without access to running wheels. The estimated variance (the amount the heart rate or ABP changes per unit of time) in ABP and heart rate was examined in Cx36-KO mice compared to WT mice using Microsoft Excel (Microsoft, Redmond, WA, USA).

Peripheral chemoreceptor reflex responses were analyzed as the peak increase or decrease in ABP and the greatest decrease in heart rate after NaCN injection and are presented as percentage change from baseline values. The magnitude of change in cardiovascular parameters (heart rate and ABP) in response to NaCN administration was compared to baseline levels and expressed as percentage change from baseline values.

### Working heart–brain stem preparation

#### Surgical procedures

Cx36-KO mice and WT littermates of either sex, between the ages of 4 and 5 wk, were pretreated with heparin sodium salt (6.2 mg/ml, i.p.; Alfa Aesar, Lancaster, United Kingdom) before being deeply anesthetized with halothane. Working heart–brain stem preparations (WHBPs) were surgically prepared as previously described (10, 11); briefly, after submerging the head and thorax in ice-cold artificial (a)CSF, decerebration at the precollicular level was followed by skinning; then the phrenic and sympathetic nerves were isolated and the diaphragm, lungs, and surrounding organs removed. After removal to a recording chamber, the descending aorta was cannulated and perfused retrogradely with aCSF.

#### Nerve recordings

Signals were recorded using glass suction electrodes attached to a head stage (NL100; Digitimer, Welwyn Garden City, United Kingdom) and fed into a Neurolog amplifier (1–2K amplification; NL104; Digitimer). Signals were passed through a Humbug (Quest Scientific, North Vancouver, BC, Canada) to filter out mains noise at 50/60 Hz. Recordings were sampled at 8 kHz and bandpass filtered between 50 Hz and 4 kHz. All recordings were digitized using an interface [CED 1401; Cambridge Electronic Design (CED), Milton, Cambridge, United Kingdom] to be analyzed with Spike2 software (CED) off-line. The phrenic nerve was cut at the level of the diaphragm and bursts of phrenic nerve discharge (PND) recorded from the central end. Central SNA was recorded from the lower thoracic sympathetic chain using a second glass suction electrode.

#### Autonomic reflexes

Peripheral chemoreceptors were stimulated with NaCN (0.03%; 0.1 ml diluted in 0.9% NaCl solution) injected directly into the descending aorta *via* the side arm of the perfusion cannula. The baroreceptor reflex was stimulated by transiently increasing perfusion flow rate. Noxious stimulation was applied to the forelimbs using manually operated hemostats for 2 s.

#### Solutions

aCSF solution contained (in mM): 125 NaCl, 24 NaHCO_3_, 5 KCl, 2.5 CaCl_2_, 1.25 MgSO_4_, 1.25 KH_2_PO_4_, and 10 d-glucose. In addition, 1.25% Ficoll (type 70; Sigma-Aldrich, St. Louis, MO, USA) was added to the perfusate; pH was measured at 7.35 ± 0.05.

#### Analysis

Stable periods of baseline PND were amplified and filtered, then integrated and rectified (time constant of 100 ms). PND frequency (bursts per minute) was calculated over at least 50 respiratory cycles during baseline recordings. The PND was calculated as the sum of the total inspiration duration and total expiration duration. Heart rate (bpm) was deduced from the electrocardiogram. During autonomic interventions, 3 trials per preparation were averaged. During each intervention, the PND rate and heart rate were analyzed immediately after the stimulus for 5 to 7 respiratory cycles. These data were then expressed as a percentage change from baseline values. Alterations in sympathetic activity were analyzed by calculating the average rectified and integrated sympathetic nerve discharge (∫SND) (with a time constant of 100 ms) during autonomic reflex stimulation and compared against the average of 2 equivalent control periods of ∫SND before and after the stimulus. This was performed using a custom-written Spike2 script, described previously (12). Because we were measuring changes in sympathetic activity, not baseline or absolute levels, noise levels were not subtracted during the analysis. The electrocardiogram was graphically removed from some traces, where appropriate, to allow clarity.

To analyze respiratory-related sympathetic bursts, the amplitude of rectified and integrated ∫SND for the first 200 ms of the PND (start of the augmenting phase, considered early inspiration) was measured and compared to the ∫SND amplitude for the first 200 ms of silencing of the phrenic nerve. These 2 amplitudes were expressed as a ratio to give a measure of the degree of respiratory-related ∫SND in the 2 conditions.

The degree of bradycardia was measured as the percentage change in heart rate (bpm) from baseline heart rate to the lowest level of heart rate after chemoreceptor stimulation. Unless otherwise stated, data are presented as group means ± sem, and differences were considered significant at the 95, 99, and 99.5% confidence limit. Statistical significance is represented as *P* < 0.05, *P* < 0.01, and *P* < 0.005, respectively. Data were tested for significance as stated within the results; *n* represents the number of preparations.

### Electrophysiology

Neonatal (7–14 d) mice of either sex, Cx36 KO, WT, or heterozygous, were deeply anesthetized with sodium pentobarbital (60 mg/kg, i.p.). An ear sample was taken for *post hoc* genotyping beforehand, and the experimenters were blinded to the genotypes of the mice during the recordings and for analysis.

Transcardial perfusion with ice-cold sucrose aCSF containing (mM) 217 sucrose, 26 NaHCO_3_, 3 KCl, 2 MgSO_4_⋅7H_2_O, 2.5 NaH_2_PO_4_, 10 glucose, and 1 CaCl_2_, buffered with 95% O_2_–5% CO_2_, was carried out. The animal was decapitated and the thoracic spinal cord removed and cut (300 μm) on a vibrating microtome (13), then placed in an immersed holding chamber containing aCSF composed of (mM) 124 NaCl, 26 NaHCO_3_, 3 KCl, 2 MgSO_4_⋅7H_2_O, 2.5 NaH_2_PO_4_, 10 glucose, and 2 CaCl_2_, equilibrated with 95% O_2_–5% CO_2_.

IML neurons were visually targeted and identified as previously described (13). Patch electrodes with resistances of 4 to 7 MΩ were filled with intracellular solution containing (mM) 110 K-gluconate, 11 EGTA, 2 MgCl_2_·6H_2_O, 0.1 CaCl_2_, 10 HEPES, 2 Na_2_ATP, and 0.3 NaGTP, pH 7.2, 285 to 290 mOsm. The voltage-gated sodium channel blocker QX-314 bromide (2–4 mM; Ascent Scientific, Bristol, United Kingdom) was included to block action potentials in the recorded neuron, thus unmasking the underlying coupled activity. The tracers neurobiotin (0.5%) and dextran–rhodamine (0.02%) were also included to allow *post hoc* morphologic analysis. These diffused into the neuron during recording with QX-314–bromide, abolishing evoked action potentials within 5 min of going whole cell.

Hyperpolarizing and depolarizing current pulses (±10–100 pA, 1 s duration, 0.14 Hz) were used to characterize recorded neurons as either SPN or interneurons on the basis of their responses (13). Neurons were held at −50 mV for the duration of the experiment. Drug solutions were bath applied (3–5 ml/min) for a minimum of 5 min. The drug used was 5-hydroxytryptamine (5-HT, 10 μM; Sigma-Aldrich) dissolved in aCSF.

#### Recovery of filled neurons

The electrode was carefully withdrawn from the slice. Slices were transferred on a slide to an epifluorescent microscope to image dextran–rhodamine fluorescence before fixation or were fixed with 4% paraformaldehyde + 0.25% glutaraldehyde overnight at 4°C when only neurobiotin was present. After resectioning at 50 µm, neurobiotin was visualized using extravidin peroxidase (1:250) and 3,3′-diaminobenzidine staining.

#### Analysis

Data were acquired at 10 kHz, filtered at 3 kHz, and logged into a computer using Spike2 v.4.1 software (CED) through a 1401plus A/D converter (CED). Off-line analysis was performed using Spike2 v7, and “spikeletlike” events were evaluated and quantified using Mini Analysis (Synaptosoft, Decatur, GA, USA). Statistical analysis was performed by Prism 5 software (GraphPad Software, La Jolla, CA, USA); results are provided as means ± sem where appropriate.

## RESULTS

### Spinal expression of CFP in Cx36-KO reporter mice

**Figure 1.**
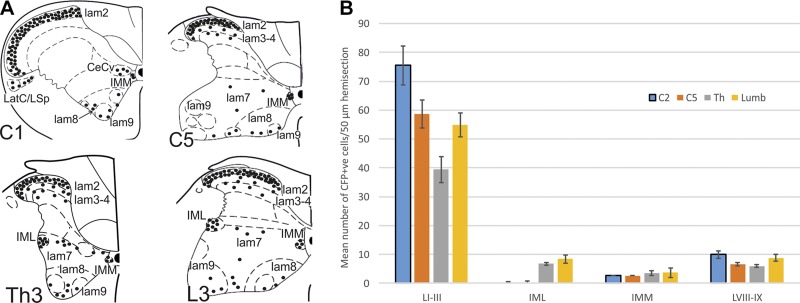
Distribution of CFP-expressing neurones in spinal cord. *A*) Representative hemisections of spinal cord at different segmental levels; each circle represents a single CFP-positive neuron in a single 50-μm section. *B*) Average number of CFP-labeled neurons in single 50-μm sections ± se from different spinal cord segments. LI–LIII, laminae I–III; IMM, intermediomedial nucleus; LVIII–IX, laminae VIII–IX.

**Figure 2.**
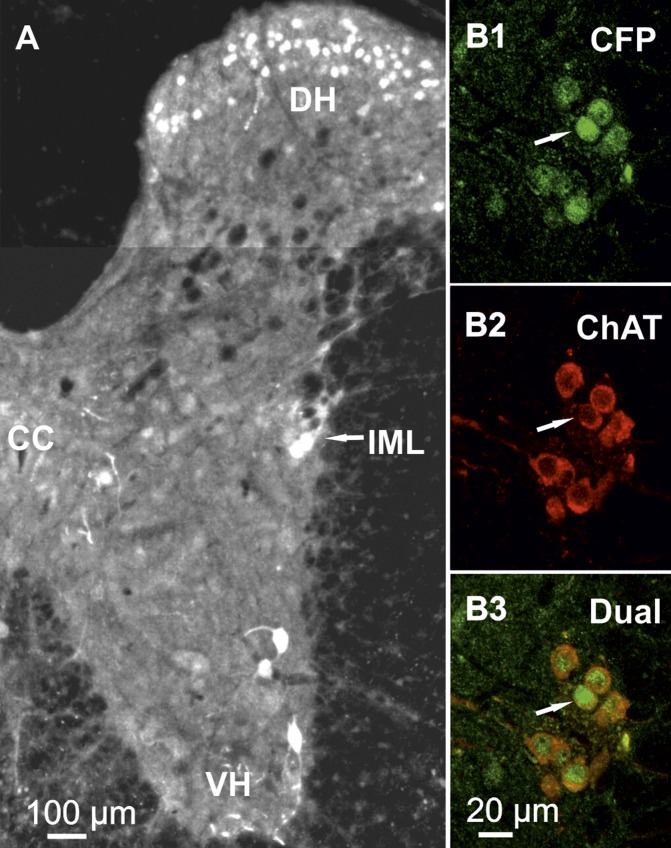
SPNs express CFP. *A*) Photomontage of thoracic spinal cord hemisection illustrating examples of CFP-labeled neurons. Grayscale images were inverted and brightness/contrast adjusted to facilitate visualization of labeled neurons. CC, central canal; DH, dorsal horn; VH, ventral horn. *B1–B3*) Example of neurons in IML expressing CFP (*B1*) and ChAT (*B2*) and combined to illustrate dual labeling (*B3*). Single-scan confocal slice; arrow indicates exemplar cell in all images.

Cells harboring the Cx36 deletion express the reporter protein CFP, enabling localization of Cx36-expressing cells. In Cx36-KO mice, numerous Cx36-expressing neurons were found in each spinal cord segment analyzed, from the first cervical to the third lumbar segment (*n* = 9 mice; **Figs. 1*A*** **and** **2**). Analysis of the distribution of cells in 50-μm spinal hemisections (Fig. 1*B*; *n* = 3 mice, *n* = 6–11 sections) revealed that the largest percentage of CFP-expressing cells was located in the superficial dorsal horn (C2, 85.5%; C5, 86.2%; T2/3, 70.7%; L3/4, 72.3%) and that the average number of cells in the dorsal horn varied with spinal cord segment (Fig. 1*B*). Fewer cells were labeled in the ventral horn, intermediomedial, and/or central cervical nucleus and in the IML (Fig. 1*B*). To determine whether CFP-expressing neurons within the IML region were SPNs, sections were dual labeled for CFP and ChAT (a marker of cholinergic cells; Fig. 2). CFP was detected in 94% of ChAT-immunopositive neurones in the IML, indicating that they are SPNs and that Cx36 is ablated in these neurons (Fig. 2*B1–B3*).

### Free-moving Cx36-KO mice have lower resting heart rate and ABP than WT mice

**Figure 3.**
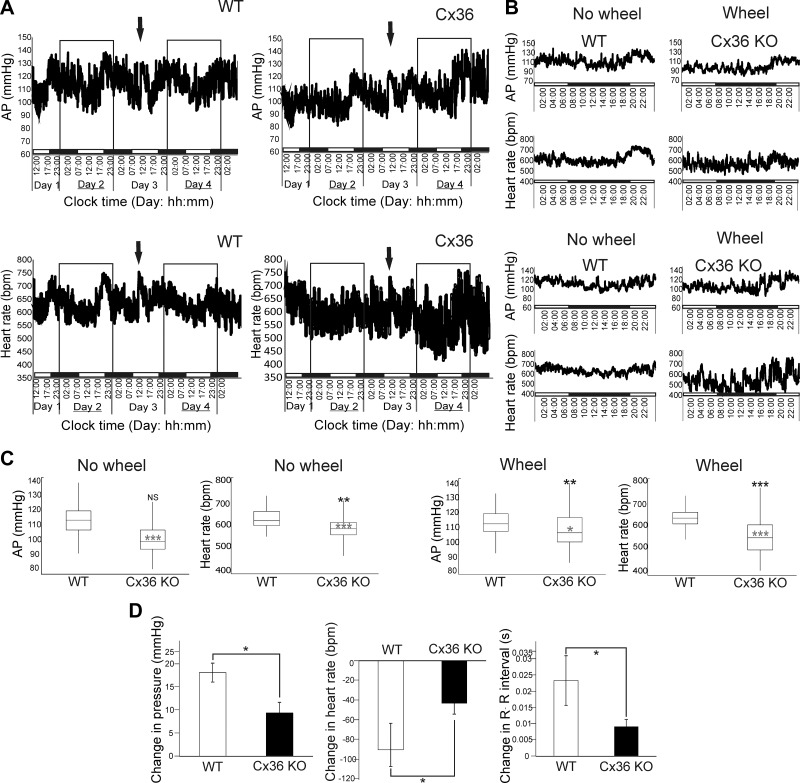
Cx36-KO mice have augmented variance in ABP and heart rate but reduced responses to chemoreceptor stimulation. *A*) Average ABP and heart rate from all animals for WT and Cx36-KO mice taken every 1 min over 5-d period. Solid arrows denote addition of free running wheel; black-and-white bar at bottom, light–dark cycle. *B*) Average data over 24-h period without running wheel (d 2) and with running wheel (d 4) as indicated by white boxes in *A*. *C*) Summary data of ABP and heart rate in Cx36-KO and WT mice, with (right) and without (left) running wheels. Variance is indicated by range of change in ABP and heart rate; black asterisks indicate significant changes in variance. Average (median) heart rate and ABP are indicated by solid line in box plots; gray asterisks denote significance. *D*) Change in ABP, R-R interval, and heart rate upon chemoreceptor stimulation in WT and Cx36-KO mice. *P < 0.05, **P < 0.01, ***P < 0.005.

Average heart rate, measured using telemetry over a 24 h period, was lower in Cx36-KO mice (582.2 ± 1.3 bpm, mean ± sem) than WT mice (629.3 ± 1.5 bpm) (*P* < 0.001, 2-sample Student's *t* test; *n* = 4; **Fig. 3*A–C***). Furthermore, heart rate variance (a statistical measure of how far values in a data set deviate from the mean) in Cx36-KO mice was significantly higher (2256.6^2^ ± 1.5) than in WT mice (1749.1^2^ ± 1.3; *n* = 4; *P* < 0.001, *F* test; Fig. 3*A–C*).

ABP was lower in Cx36-KO mice (100.6 ± 0.3 mmHg) compared to WT mice (113.6 ± 0.3 mmHg) (*n* = 4; *P* < 0.001, 2-sample Student’s *t* test; Fig. 3*A–C*); however, ABP variance was not significantly different (Cx36-KO mice, 79.5^2^ ± 0.7; WT mice, 84.8^2^ ± 0.5; *n* = 4, *P* = 0.224, *F* test; Fig. 3*A–C*).

### Free-moving Cx36-KO mice with access to running wheels display augmented variance in ABP and heart rate compared to WT mice

In Cx36-KO mice with access to running wheels, heart rate was lower (552.2 ± 2.63 bpm; WT 629.3 ± 1.1; *n* = 4; *P* < 0.001, 2-sample Student’s *t* test; Fig. 3*A–C*) while variance was significantly higher (7042.3^2^ ± 2.7) than that of WT mice (1228.9^2^ ± 1.1; *n* = 4; *P* < 0.001, *F* test; Fig. 3*A–C*). Furthermore, ABP (109.6 ± 0.3 mmHg) was lower than WT mice (114.0 ± 0.3 mmHg; *P* = 0.01, 2-sample Student’s *t* test; *n* = 4; Fig. 3*A–C*). Variance in ABP in Cx36-KO mice (118.9^2^ ± 0.3) was higher compared to WT mice (68.4^2^ ± 0.3; *n* = 4, *P* < 0.01, *F* test; Fig. 3*A–C*). Values are summarized in **Table 1**. This suggests that during exercise, the regulation of ABP is impaired in Cx36-KO mice.

**TABLE 1.** Average pressure and average heart rate in WT and Cx36-KO mice with and without access to running wheels

Wheel	WT		Cx36 KO	
	Blood pressure (mmHg)	Heart rate (bpm)	Blood pressure (mmHg)	Heart rate (bpm)
Without	113.6 ± 0.3	629.3 ± 1.5	100.6 ± 0.3***	582.2 ± 2.8***
	84.8^2^ ± 0.5*^a^*	1749.1^2^ ± 1.3*^a^*	79.5^2^ ± 0.7*^a^*	2256.6^2^ ± 1.5***^a^
With	114.0 ± 0.3	629.3 ± 1.1	109.6 ± 0.3*	552.2 ± 2.6***
	68.4^2^ ± 0.3*^a^*	1228.9^2^ ± 1.1*^a^*	118.9^2^ ± 0.3**^a^	7042.3^2^ ± 2.7***^a^

a Variance. *P < 0.05, **P < 0.01, ***P < 0.005.

### Anesthetized Cx36-KO mice exhibit attenuated heart rate responses to peripheral chemoreceptor stimulation compared to WT mice

Chemoreceptor stimulation increased ABP and elicited a pronounced bradycardia in anesthetized Cx36-KO and WT mice. In WT mice, ABP increased by 16.3 ± 1.2 mmHg; in Cx36-KO animals, this increase was attenuated compared to WT mice (9.4 ± 1.3 mmHg; *P* = 0.01, 2-sample Student’s *t* test). The R-R interval in WT mice (0.114 ± 0.001 s at baseline) was increased by 0.023 ± 0.007 s at the peak of the chemoreceptor stimulation response. In Cx36-KO mice, this increase in R-R interval was significantly smaller (*n* = 4; *P* = 0.03, 2-sample Student’s *t* test; Fig. 3*D*) such that chemoreceptor stimulation only caused an 0.006 ± 0.0002 s increase in R-R interval from a baseline value of 0.110 ± 0.006 s. Thus, bradycardia upon peripheral chemoreceptor stimulation was attenuated in Cx36-KO mice (35.9 ± 10.8 bpm) compared to WT (84.9 ± 21.8 bpm; *P* = 0.01, 2-sample Student’s *t* test; Fig. 3*D*).

### Resting heart rate and sympathetic activity are lower in the WHBP of Cx36-KO mice compared to WT mice

To investigate whether Cx36-containing GJs contribute to the regulation of SNA, direct recordings of SNA were obtained in the anesthesia-free WHBP.

**Figure 4.**
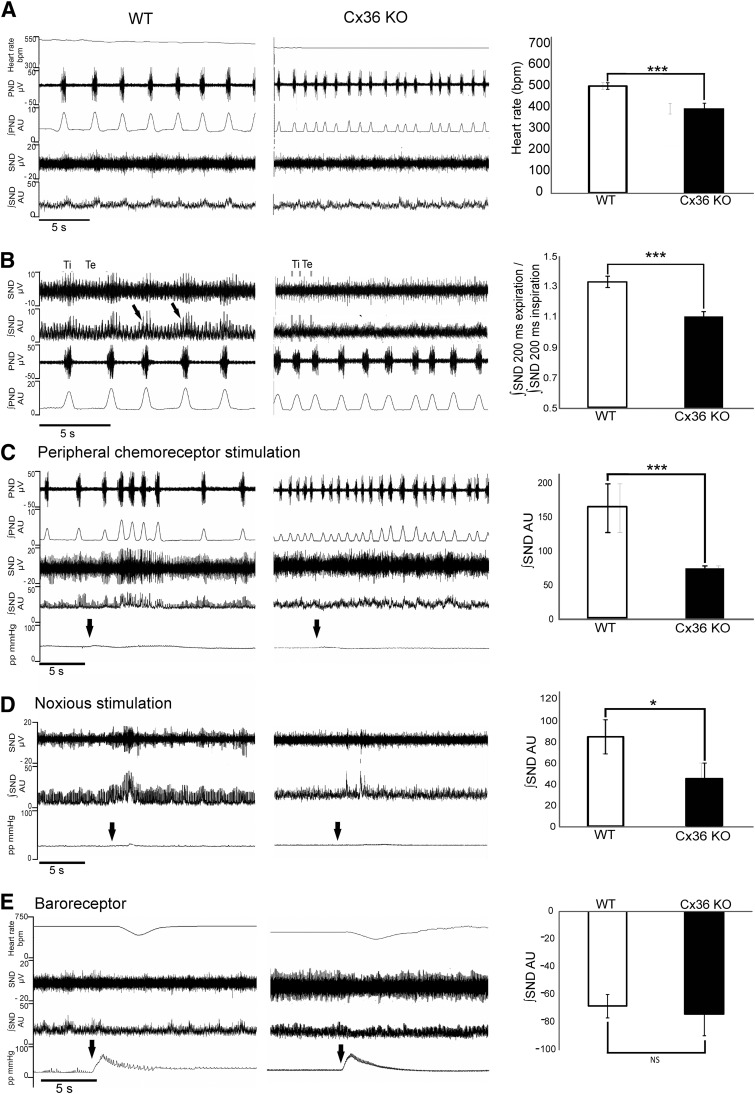
In WHBP Cx36-KO mice have reduced respiratory-related SNA and attenuated responses to chemoreceptor and noxious stimulation. *A*) Raw data from Cx36-KO mice showing heart rate, PND, and SND. On right, group data illustrate reduced resting heart rate in Cx36-KO mice. *B*) Raw data of SND and PND, with inspiratory and expiratory phases of respiratory cycle marked as total inspiration (Ti) and total expiration (Te), respectively. Group data on right shows attenuated respiratory-related SND in Cx36-KO mice. *C*) Peripheral chemoreceptor stimulation (arrows) in WT and Cx36-KO mice. Group data show that percentage sympathoexcitation upon chemoreceptor stimulation is significantly reduced in Cx36-KO mice. *D*) Noxious stimulation (arrow) elicited short latency increase in SND in both groups, but this was significantly diminished in Cx36-KO mice and is shown by group data on right. *E*) Sympathoinhibition resulting from baroreceptor stimulation was not significantly different in Cx36-KO mice compared to WT mice. *P < 0.05, **P < 0.01, ***P < 0.005.

Similar to data from conscious animals, in the WHBP heart rate was significantly lower in Cx36-KO mice (415.7 ± 25.2 bpm) than WT mice (525.2 ± 15.9 bpm; *n* = 21; *P* = 0.001, 2-sample Student’s *t* test; **Fig. 4*A***) despite a higher respiratory rate in the WHBPs of Cx36-KO (Fig. 4*A, B*). Sympathetic nerve discharge (SND) typically exhibited tonic activity with characteristic respiratory-related increases in discharge in both WT mice and in Cx36-KO mice. However, the degree of respiratory-related SND was 19.2% smaller in Cx36-KO mice compared to WT mice. The ratio between the SND during the first 200 ms of expiration and the first 200 ms of inspiration was 1.0 ± 0.1 in WT mice, while in Cx36-KO mice the ratio decreased to 0.8 ± 0.0 (*n* = 6; *P* = 0.02, 2-sample Student’s *t* test; Fig. 4*B*).

### Using the WHBP of Cx36-KO mice, sympathetic responses to peripheral chemo- and nociceptive stimuli are smaller than those of WT mice but baroreceptor responses are similar

In Cx36-KO mice, the sympathoexcitation of 71.7 ± 60.1 arbitrary units (AU) (*n* = 12) in response to peripheral chemoreceptor stimulation (0.03% NaCN injected into the descending aorta *via* the side arm of the perfusion cannula) was significantly smaller than that observed in WT mice (163.3 ± 36.1 AU; *n* = 15; *P* = 0.001, 2-sample Student’s *t* test; Fig. 4*C*), although differing baseline levels of SND in WT and KO may have influenced the amplitudes of these responses. The average bradycardia upon chemoreceptor activation was lower in Cx36-KO mice (141.7 ± 17.4 bpm) than in WT mice (211.8 ± 25.1 bpm; *n* = 15; *P* = 0.033). Nociceptive stimulation (elicited to the forelimbs using manually operated hemostats for 2 s) also caused sympathoexcitation in both Cx36-KO and WT mice. However, in Cx36-KO mice, this sympathoexcitation was significantly smaller (54.9 ± 16.7 AU, *n* = 12) compared to WT mice (100.2 ± 18.5 AU; *n* = 15; *P* = 0.029, 2-sample Student’s *t* test; Fig. 4*D*).

Baroreceptor stimulation (transient increase in perfusion flow rate) resulted in a sympathoinhibition of 72.4 ± 15.0 AU in Cx36-KO mice, which was not significantly different from that of WT mice (67.6 ± 11.9 AU) (*n* = 15; *P* = 0.784, 2-sample Students *t* test; Fig. 4*E*). There was also no change in the baroreceptor reflex gain sensitivity (expressed as the change in heart rate (bpm) per 1 mmHg change in perfusion pressure) between Cx36-KO mice and WT mice (−5.9 ± 0.5 and −5.5 ± 0.6 bpm mmHg −1, respectively) (*n* = 15; *P* = 0.588, 2-sample Student’s *t* test), although it must be noted that the starting heart rate was significantly lower in the Cx36-KO animals, which may influence the results. There were no significant differences in the mean level of inspiratory related or expiratory related ∫SND between WT (*n* = 15; *P* = 0.364) and Cx36-KO mice (*P* = 0.140, 2-sample Student’s *t* test; *n* = 15).

### SPN recordings in spinal cord slices show lower levels of spikelet activity in Cx36-KO mice compared to WT mice

To determine whether GJ coupled activity between SPNs is reduced in Cx36-KO mice, single recordings from SPNs were made in spinal cord slices from WT and Cx36 heterozygous and KO mice.

**Figure 5.**
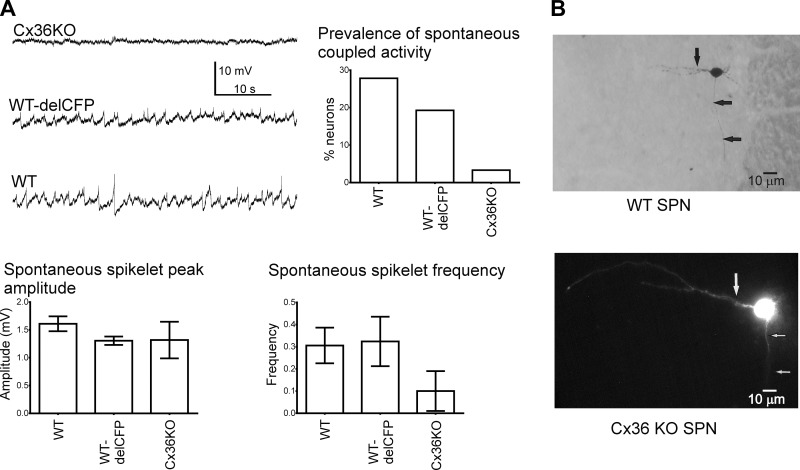
Spikelet frequency in SPNs is reduced in Cx36-KO mice. *A*) Representative traces taken from single SPNs (held at −60 mV) recorded in spinal cord slices of Cx36-KO, heterozygous (Cx36^+/delCFP^), and WT mice showing lack of ongoing spikelet activity in Cx36-KO compared to other 2 groups. Histogram to right is grouped data showing prevalence of spikelets in SPNs from 3 groups. In those 2 SPNs in Cx36-KO mice where spikelets were observed, neither frequency nor amplitude of spikelets was significantly different from heterozygous or WT animals. *B*) Typical morphology of SPN in WT. Note axon heading ventrally and mediolaterally orientated dendrites (arrows).

SPNs from WT (*n* = 79), heterozygous (Cx36^+/delCFP^, *n* = 83), and Cx36-KO (*n* = 60) mice were recorded. These cells were characterized as SPN on the basis of a combination of their anatomic location and responses to positive and negative current pulses, and these did not differ between mouse genotypes, as expected from previous studies (14). Because the majority of SPNs (94%) in the IML are CFP positive, they were not verified for CFP in Cx36-KO slices during these experiments. Those cells imaged using dextran–rhodamine or diaminobenzidine in both WT and Cx36-KO mice displayed the characteristic mediolateral orientation of SPNs with somata located within the IML, medially projecting dendrites, and ventrally projecting axon (**Fig. 5*B***). This similar morphology fits with recent observations that in older mice, there is no significant difference in number or length of primary dendrites between Cx36-KO and WT mice (15). Consistent with previous observations in rat SPNs (16), spontaneous coupled activity in the form of spikelets was observed in 27.8% of WT SPNs held at −50 mV (*n* = 22/79; Fig. 5*A*), with a mean frequency of 0.3 ± 0.08 Hz (*n* = 16). Neither prevalence nor frequency of spontaneous spikelet activity were significantly different in heterozygotes (*n* = 16/83, 0.3 ± 0.11 Hz) compared to WT mice. Importantly, most SPNs (97%) in the Cx36-KO mice did not exhibit spikelets; prevalence was thus significantly lower compared to both WT and heterozygotes (*P* < 0.0001, Fisher’s exact test; Fig. 5*A*). Furthermore, in those 2 SPNs where some spikelets were observed, their frequency was significantly lower (0.1 ± 0.09 Hz) in Cx36-KO compared to WT mice.

**Figure 6.**
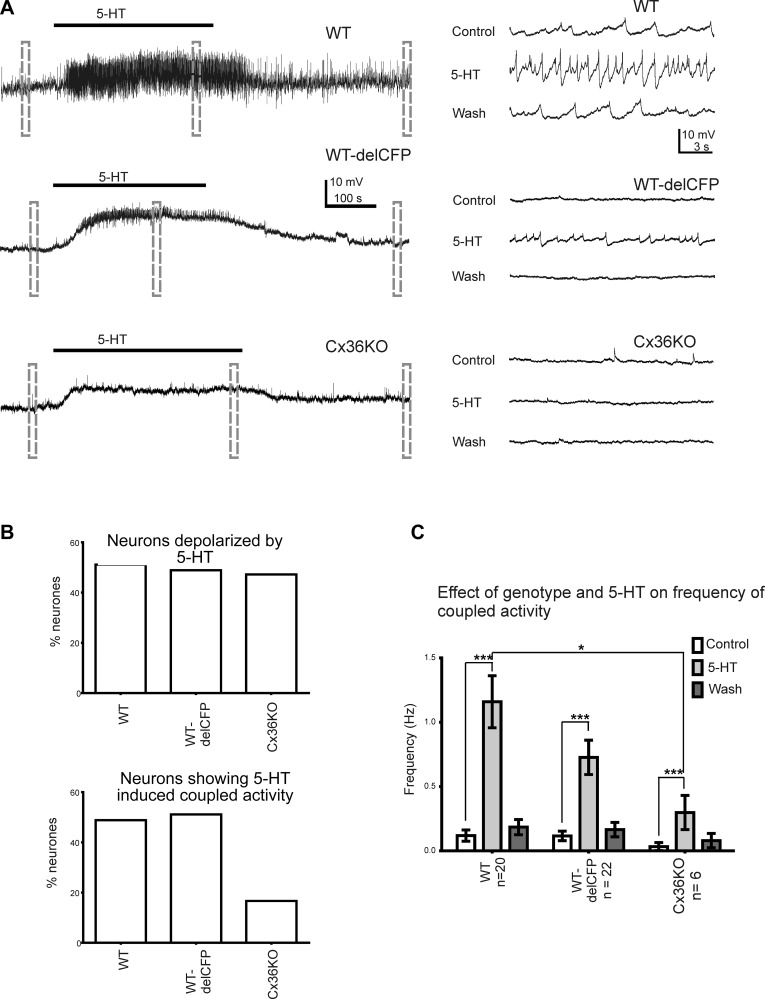
5-HT-induced spikelet activity is significantly attenuated in Cx36-KO mice. *A*) Representative traces taken from single SPNs in spinal cord slices of Cx36-KO, heterozygous (Cx36^+/delCFP^), and WT mice showing responses to bath application of 10 µM 5-HT (black line denotes time of application; negative current was added to bring resting membrane potential to initial starting value of −60 mV). Dashed lines show areas of trace that are shown at right on faster time base to clearly see spikelets. *B*) Pooled data of percentage of SPNs in 3 groups that were depolarized by 5-HT (top) and percentage that showed 5-HT induced coupled activity (bottom). *C*) Pooled data from 3 groups showing effect of 5-HT on frequency of spikelets only in SPNs that responded with change in spikelet occurrence or frequency.

Application of 5-HT has excitatory effects on SPNs, which includes increasing the prevalence of spikelets (16). As most SPNs are quiescent, 5-HT (10 μM) application was used to induce spikelets in these cells. Depolarization in response to 5-HT was equally prevalent between WT (51.2%), heterozygote (48.9%), and Cx36-KO (47.2%) SPNs, with no observable difference in amplitude of depolarization (6.8 ± 1.0 mV WT; 5.6 ± 1.0 mV, heterozygotes; 5.9 ± 1.2 mV, Cx36 KO; *P* > 0.7; **Fig. 6*A, B***). This is in contrast to the induction or augmentation of spikelet activity in the presence of 5-HT, which remained equally inducible in WT (48.8%) and heterozygotes (51.1%), but the percentage of SPNs in Cx36-KO animals that responded to 5-HT with spikelets was significantly lower (16.7%; Fig. 6*A, B*) than the other groups. The effect of 5-HT on spikelet frequency in responsive SPNs remained significant (*P* < 0.001, 2-way ANOVA) across all genotypes, but there was a significant reduction in mean frequency between WT (1.16 ± 0.2 Hz, *n* = 20) and Cx36-KO (0.298 ± 0.13 Hz) mice (*P* < 0.05, 2-way ANOVA, Fig. 6*C*).

## DISCUSSION

This study is the first to show that KO of the Cx36 GJ protein results in abnormal physiologic regulation of ABP and heart rate in freely moving mice. Utilizing the WHBP preparation revealed that Cx36 KO disrupted SNA, heart rate, and ABP. A reduction in spikelet activity (which is considered to be due to GJ coupling between SPNs (17)) was observed between SPN of Cx36-KO mice. Together, these results reveal reduced sympathetic activation and a loss of temporal coordination of this activity upon loss of Cx36.

### Cx36-containing GJs preferentially influence sympathetic autonomic activity

KO of Cx36 preferentially affects sympathetic over parasympathetic nervous activity. The vagal outflow to the heart was unaffected by Cx36 KO because baroreceptor reflex sensitivity did not change. However, converging observations in the Cx36-KO mice indicate Cx36 influence on the sympathetic nervous system are: *1*) significantly increased variability in ABP is consistent with an impaired sympathetic baroreflex; *2*) a blunted reflex bradycardia after chemoreceptor stimulation is due to initial vagal activation but with a contribution from sympathetic withdrawal (18); and *3*) attenuation of sympathoexcitation upon peripheral injections of NaCN or to noxious stimulation. It must be noted that baseline parameters are changed in the Cx36-KO animals, which may influence the amplitudes of the responses observed to baroreceptor, chemoreceptor, or noxious stimulation. However, similar blunted chemoreflex responses are observed in patients with multiple system atrophy where there is a profound loss of both pre- and postganglionic neurons (19, 20).

The absence of Cx36 therefore has major consequences on central sympathetic drive; this is consistent with the resting bradycardia observed in Cx36-KO mice, which is unlikely to be due to direct effects on the heart as neuronal Cx36 is not expressed here (21). Because chemical thoracic sympathectomy results in a pronounced bradycardia, this suggests that reduced sympathetic activity can indeed have depressant effects on heart rate (22). Furthermore, this bradycardia is consistent with data from patients taking mefloquine (which affects Cx36 GJs) for malaria prophylaxis (23); the fact that this drug crosses the blood–brain barrier (24) may mean that the bradycardic effects observed are due at least in part to loss of Cx36-containing GJs, resulting in disrupted central sympathetic activity. The bradycardia may be due to sympathetic withdrawal *via* changes in GJ coupling in the central connections between the nucleus of the solitary tract, the ventrolateral medulla, and SPNs, but a major likely site is at the level of the SPNs themselves. It could be also argued that the bradycardia and reduced blood pressure observed in Cx36-KO mice may be due to reduced physical activity of these mice associated with loss of Cx36 in motor circuits rather than specific loss of Cx36 at the level of SPNs innervating these cardiovascular targets. There are, however, no studies indicating reduced locomotor activity in Cx36-KO mice (25).

### Loss of Cx36-containing GJs in the SPNs may underlie some of the effects observed

The Cx36-KO model used here is a global KO, and the effects observed may thus be due to loss of GJ function at a number of sites. For example, Cx36 is expressed in the pancreas, although Cx36-KO mice have normal plasma insulin and glucagon levels and no difference in fasting glucose levels (26), so it is unlikely that this would have a significant effect on sympathetic activity. One likely locus for at least some of the effects of KO of Cx36-containing GJs on SNA is SPNs. SPNs in the KO mice express CFP, which indicates Cx36 expression. In addition, in spinal cord slices, the incidence of spikelets is much reduced in SPNs recorded from Cx36-KO mice, although not completely abolished, similar to that observed in thalamic reticular nucleus neurons (15). This suggests that a potential site of action for the altered cardiovascular responses in the Cx36 KO is *via* the GJs between SPNs. This is further supported by previous data using paired recordings that demonstrate synchronization of action potential firing and spikelets between electrotonically connected SPNs (17). Furthermore, rhythmic network activity recorded from the IML is reduced with the broad spectrum GJ blocker carbenoxolone and can be abolished by the Cx36 GJ blocker mefloquine (6). We do not rule out the possibility that some of the changes in cardiovascular and sympathetic variables observed in our study are due to loss of Cx36 in other neurons (potentially including those in the sympathetic ganglia) that form part of the sympathetic circuits underlying cardiovascular control (27). However, it is likely, both from our results and from previous research (8), that at least one factor underlying the changes is SPN-specific loss of Cx36-containing GJs. To address this directly would require targeted KO of SPNs in future studies once such transgenic models for targeting SPNs are available.

The sympathetic nervous system requires coordination of functionally related activity across a large column of neurons, and GJ expression may be consistent with this functional specialization of sympathetic outflow. GJ expression between SPNs with the same autonomic functions may play an important role in coordinating the activities of different neurons involved in the same sympathetic response. In this way, GJs may coordinate populations of neurons to produce a synchronized response rather than increasing basal levels of SND. In spinal cord slice preparations, only about a quarter of SPNs exhibit GJ coupling in the form of spikelets (17), which would indicate some degree of functional specificity; recent evidence using the WHBP has supported such a suggestion (28).

We found that there were still some incidences of spikelet activity in Cx36-KO animals, in keeping with previous reports using these KO animals where some coupled activity was preserved (15) or when dye coupling was observed in a small percentage (14%) of thalamic reticular nucleus neurons (29). Despite use of neurobiotin in the recording electrode, we did not observe dye coupling in SPNs in either the WT or KO mice, regardless of the presence of spikelets. Similar lack of dye coupling in these spinal cord neurons has been reported previously (17).

### Functional GJ expression in adult rodents

There is still debate over the contribution of GJs to SPN coordinated activity in adults because some consider that GJ expression in these neurons is a developmental phenomenon (5) and thus may not contribute to sympathetic outflow in adults. However, the Cx36 protein is immunohistochemically detected in SPNs in adult rat (8), and pharmacologic blockade of Cx36-containing GJs influenced sympathetic variables in rats aged 4 to 6 wk (10). Furthermore, GJ coupling between SPNs in adult spinal cord slices was reported in a brief communication (30), while in the WHBP of increasing age from P5 to P16, there is no reduction in the number of SPNs that exhibit spikelets (28). This current study supports this evidence because Cx36 KO alters cardiovascular regulation in adult mice. This may also apply to humans, as patients taking the Cx36 blocker mefloquine as an antimalarial agent exhibited a significant bradycardia 6 d after administration (23). Cx36-mediated GJ therefore appears important for central control of the cardiovascular system in adult mammals.

### Functional significance of findings

Chronically elevated levels of SNA are associated with disorders including heart failure, obesity, obstructive sleep apnea, and hypertension (31). Evidence to support a role for the sympathetic nervous system in controlling ABP is well documented; chemical sympathectomy in unanesthetized rats significantly increased ABP variability (32, 33). Our data indicate that Cx36 is likely involved in the precise regulation of SNA (and thus heart rate and ABP) during periods of rest, activity, or specific perturbation, such as noxious stimulation. Indeed, in Cx36-KO mice, the variance in heart rate and BP resulting from the introduction of a running wheel was at least twice that of WT. Loss of Cx36 may result in decreased vasomotor tone and a loss of precise control of cardiac output or vasoconstriction by the SPNs acting on the heart and blood vessels. Means to modulate GJs may therefore be a welcome noninvasive therapy for restoring the sympathovagal balance that underlies many disorders.

## Abbreviations

5-HT: 5-hydroxytryptamine
ABP: arterial blood pressure
aCSF: artificial cerebrospinal fluid
AU: arbitrary units
bpm: beats per minute
CFP: cyan fluorescent protein
ChAT: choline acetyltransferase
Cx36: connexin 36
GFP: green fluorescent protein
GJ: gap junction
IML: intermediolateral cell column
∫SND: integrated sympathetic nerve discharge
KO: knockout
PGK: phosphoglycerate kinase I promoter
PND: phrenic nerve discharge
R-R: interbeat interval
SNA: sympathetic nerve activity
SND: sympathetic nerve discharge
SPN: sympathetic preganglionic neuron
WHBP: working heart–brain stem preparation
WT: wild type
